# CNS fluid and solute movement: physiology, modelling and imaging

**DOI:** 10.1186/s12987-020-0174-1

**Published:** 2020-02-04

**Authors:** Hazel C. Jones, Richard F. Keep, Lester R. Drewes

**Affiliations:** 1 Gagle Brook House, Chesterton, Bicester, OX26 1UF UK; 2 Department of Neurosurgery, University of Michigan, Ann Arbor, MI 48105 USA; 3 Department of Biomedical Sciences, University of Minnesota Medical School Duluth, Duluth, MN 55812 USA

In 2018, we invited readers of *Fluids and Barriers of the* CNS to contribute to a Thematic Series on aspects of fluid and solute movement into, within, and out of the brain. This subject has important implications for neurological diseases such as stroke, Alzheimer’s disease (AD), idiopathic intracranial hypertension (IIH), normal pressure hydrocephalus (NPH) and syringomyelia. It is also an important factor in the delivery and distribution of pharmaceutics for disease treatment.

The submitted papers covered a variety of themes. One focused on cerebrospinal fluid (CSF) secretion or flow within the ventricles and subarachnoid spaces (SAS). Another theme centred on the controversial subject of perivascular flow around penetrating blood vessels using mathematical modelling. A third concerned movement of fluid and solutes through the brain interstitial space: a topic of great interest but difficult to study experimentally.

Advances in imaging have provided new insights into fluid and solute movement in the CNS. Techniques for improved resolution in magnetic resonance imaging (MRI) are enabling a better understanding of factors affecting CSF flow. For example in normal subjects, forced abdominal breathing had a larger effect on CSF flow than thoracic breathing especially in the spinal cord SAS, with inspiration resulting in upward flow [1]. Another study [2] demonstrated that in the cerebral aqueduct, the cardiac pulse had a larger effect on CSF velocity than the respiratory pulse, but the reverse was true for displacement volume. These studies in normal subjects can set the baseline for improved diagnosis of abnormal CSF dynamics in disease. For example, flow velocity and displacement volume were both increased in the aqueduct of a group of NPH patients but were reduced after shunt surgery [3]. Another study [4] found that both NPH and AD patients had similar but abnormal aqueduct CSF pressure gradients. However, the velocity was very variable in NPH patients and not abnormal in AD. After a series of consecutive lumbar drains, CSF compliance and cerebral perfusion pressure increased in IIH patients with raised intracranial pressure [5]. Although this invasive technique precludes a study with control subjects for comparison, it could be extended to predict treatment response. Another promising approach for modulating CSF dynamics in disease is to target secretory mechanisms in the choroid plexus, although this becomes very complex when considering the number of potential targets [6], especially when a large number of genes were shown to be altered in choroid plexus tissue from AD patients [7].

In vitro models of the CSF system are also being used increasingly to delineate CSF dynamics and to study pathological conditions. A 3D-model of the SAS was constructed from meningoepithelial cells and exposed to different flow conditions [8]. Abnormal CSF flow and hypoxia resulted in significant changes in expression of genes from the cultured cells involved in extracellular matrix composition, the endosome-lysosome system, and mitochondrial energy metabolism. A constructed model has been developed to investigate CSF flow and compliance during pathological disturbances [9], and a mathematical model was used to predict that jugular vein collapse reduces the fall in intracranial pressure when moving to upright posture and makes a significant contribution to mitigate the postural increase in intracranial compliance [10]. To assist research into diagnosis and the administration of therapeutics, a 3D geometric and hydrodynamic model of the spinal subarachnoid CSF was constructed from high resolution MR images of the entire spine of a healthy subject and is available for reuse under license [11]. This model has been used to investigate the dynamics of spinal CSF and potential for intrathecal therapy in patients with amyotrophic lateral sclerosis [12].

The removal of metabolic products and toxic substances from the brain interstitial space is important for neuronal health and a subject of intense research. Small solutes, transporter substrates and lipid soluble substances can be eliminated across the blood–brain barrier/neurovascular unit (BBB/NVU), whereas large polar molecules including amyloid-β may, at least in part, be eliminated via the perivascular spaces (reviewed in [13]). It has long been known that tracers in the SAS may enter brain parenchyma around penetrating blood vessels, particularly arteries/arterioles [14], although there were questions over the rate and significance of such movement [15]. Similarly, there has long been evidence that tracers placed in brain parenchyma may exit the brain to the CSF via the perivascular space [16]. Two different potential routes for fluid/solute movement around cerebral blood vessels have been identified, a space between the pia and astrocyte end feet (paravascular) and a route along the basement membranes of the smooth muscle layer (perivascular) [17–19]. This has raised many questions as to the physiological implications of these observations, not least because the movement of marker molecules does not necessarily have to occur by bulk fluid flow (convective flow). One suggestion is that CSF entering the brain via paravascular channels moves through the brain parenchyma via an astrocyte aquaporin-4 dependent pathway and clears the brain of waste products by exiting via paravenous channels (the glymphatic hypothesis [20]). However, this is still the subject of much debate [21, 22].

Injection of a fluorescent tracer into rat spinal cord showed that radial spread occurred within the parenchyma from grey matter into white matter but not vice versa, and most importantly tracer was seen in the paravascular spaces of arteries, arterioles and venules and also in the arterial perivascular space [23]. In the opposite direction, constriction of the rat spinal cord SAS at the cervicothoracic junction followed by intracisternal injection of fluorescent ovalbumin resulted in fluorescence in the parenchyma and also around arterioles, venules and capillaries, effects seen in control and to a greater extent in constricted animals [24]. Using data from a previous study [25] in which MR contrast agent injected intrathecally was localised in the CSF and brain by sequential imaging, convection and diffusion in the parenchyma was modelled using uncertainty quantification [26]. It was concluded that uncertainty in the diffusion coefficient was not sufficient to account for the tracer movement into white matter and that the addition of a convective velocity field (glymphatic?) may be needed to explain the data.

The evidence for fluid movement in spaces around brain vessels raises the question of the driving force, and potential mechanisms have been considered using mathematical modelling. The cardiac pulse in arterial vessels has been considered as a mechanism for propelling fluid and solutes in spaces around vessels. However, a hydraulic network model using parameters from the literature concluded that oscillatory fluid motion does not result in perivascular net flow but that solute movement may be enhanced by dispersion [27]. The hydraulic resistance of periarterial, para-arterial and para-venous channels has also been estimated using a simplified model of the cerebral vascular tree and it was concluded that the resistance was too high to allow for pressure-driven flow in any of the potential routes [18]. However, another study has shown that the shape of the periarterial space (concentric and circular or elliptical) has a large effect on the estimated hydraulic resistance and may explain some discrepancies [28]. Shear-augmented dispersion of solutes has been considered as an alternative a mechanism for solute movement using a mathematical model which takes into account the nature of the medium (porous or non-porous) and the pulsatile movement of fluids. It was concluded that such augmentation is unlikely in basement membranes but could be important in 10 µm para-arterial spaces and also in the spinal SAS [29]. Readers are also referred to recent reviews that have addressed these points [30, 31].

Mechanisms by which solutes move in the interstitium of the parenchyma are also a focus of much research (see recent review [32]). A hydraulic model predicted that in the parenchyma solute movement occurs largely by diffusion [27]. A different approach to estimating interstitial flow was taken by Ray et al. [33], using data from iontophoretic infusion of a small ionic molecule with concentration measurement at a known distance from the infusion. From the simulations, they concluded that both diffusion and bulk flow may be important, with bulk flow more important for large molecules. However, the calculations for flow in this study have been questioned [34] (see [35] for response). While the movement of fluid and solutes within the interstitium is important for a more complete understanding of brain physiology and pathophysiology, it may also impact drug delivery for neurological diseases. A review has considered a number of different published models for predicting drug distribution and concluded that they are incomplete. Transport across the BBB/NVU, movement within the brain, and molecular binding all need to be taken into account to create a 3D model that predicts drug concentration in time and space [36].

Debates over the glymphatic system and CSF production and flow have made brain fluid and solute dynamics an area of intense research. It is a very important topic influencing normal brain function and disease states, as well as drug delivery. The papers in this Thematic Series reflect that importance and the variety of approaches that are being used to address it.
